# Potential signaling pathways, biomarkers, natural drugs, and chronic myeloid leukemia therapeutics

**DOI:** 10.3389/fphar.2025.1615770

**Published:** 2025-10-27

**Authors:** Sultan Alqahtani

**Affiliations:** ^1^ Department of Basic Medical Sciences, College of Medicine, King Saud bin Abdulaziz University for Health Sciences, Riyadh, Saudi Arabia; ^2^ King Abdullah International Medical Research Center (KAIMRC), Riyadh, Saudi Arabia

**Keywords:** potential signaling pathways, biomarkers, chronic myeloid leukemia (CML), disease progression, myeloid sarcoma, CML therapeutics, natural drugs

## Abstract

The Philadelphia chromosome signals BCR-ABL1 migration in myeloid clonal proliferation disorders such as chronic myeloid leukemia (CML). The crucial function of the Musashi2-Numb axis in deciding cell fate and its connection to significant signaling pathways like Hedgehog and Notch, which are necessary for the self-renewal pathways of CML stem cells, will be the subject of future research in this work. For this review, we conducted a PubMed search using the terms Musashi2-Numb, signaling pathways, and leukemia. As a result, we assembled several studies. Tyrosine kinase inhibitors like imatinib can kill and eradicate BCR-ABL1 translocated cells, but they cannot directly target BCR-ABL1 leukemia stem cells. The primary issue is stem cells’ resistance to imatinib therapy. Since leukemia stem cells are thought to be treated by the Musashi2-Numb signaling pathway, a successful therapy approach may involve comprehending and controlling the downstream molecules and signaling pathway of BCR-ABL1 that are important in the survival and self-renewal of leukaemia stem cells. Here, we focused on the generalised perspectives of the drugs that target major signaling proteins and change elements or pathways downstream of BCR-ABL1 can effectively treat chronic leukemia stem cells. There are handful number of proteins such as Musashi2 which have substantial diagnostic use in leukemia treatment and strategy. After going through a number recent develeopments in CML and its therapeutics, I presented here an overview of the latest advancements in CML, natural drugs, biomarkers, potential signaling pathways, and treatment strategies.

## 1 Introduction

Abnormal cell proliferation, which primarily permits cells to penetrate and migrate from the originating location to other sections of the body, is a characteristic of a family of disorders known as cancer (Allavena et al., 2008; Hanahan, 2022; Hanahan and Weinberg, 2011; Stangis et al., 2024). Leukemia arises when bone marrow functions are disturbed and unchecked cell division takes place. Leukemia can result in early death if treatment is not received. It arises when progenitor cells, sometimes referred to as precursor cells, in bone marrow grow out of control and prevent the production of healthy blood cells. Acute and chronic leukemias are classified into lymphoid and myeloid lineages based on their origins (Ahmed and Etten, 2013; Andersson et al., 2015; Balandrán et al., 2023; Bosch and Dalla-Favera, 2019; Clarke et al., 2016; Di Giorgio et al., 2023; Golub et al., 1999).

A monoclonal, rapidly spreading cancer linked to the myeloid lineage is chronic myeloid leukemia (CML). A change between chromosomes 9 and 22 and a rise in clonal hemopoietic stem cells (HSCs) are characteristics of CML. The Philadelphia (Ph) chromosome is the end outcome. Tyrosine kinase BCR-ABL1, which is always active and the activator of several molecular pathways, is created when the breakpoint cluster region protein (BCR) gene, located in the 22q11 zone, is positioned in the inverted Abelson murine leukemia viral oncogene homolog 1 (ABL1) gene, which is located in 9p34. In the end, this gene causes aberrant cell adhesion, accelerated cell proliferation, and suppression of apoptosis. Blastic, rapid, and chronic are the three stages of CML clinical stages. The percentage of bone marrow and blood blasts increases over time, despite a gradual start. Following the expedited phase, there will be greater resistance to treatment during the blast crisis stage (Andersson et al., 2015; Balandrán et al., 2023; Bosch and Dalla-Favera, 2019; Banjar, 2018; Bawazir et al., 2019; Branford et al., 2018; Chandran et al., 2019; Clark, 2007; Clarkson et al., 2003; Druker et al., 2001a; Druker et al., 2001b; Eagle et al., 2022; Fabbri et al., 2011; Garcia‐Manero et al., 2003; Janowski et al., 2022; Jensen et al., 2013; Kennedy and Hobbs, 2018).

In numerous biological processes, including development, differentiation, metabolism, and death, tyrosine kinase (TK) is a crucial mediator of the signaling cascade (Table 1). The first molecularly targeted medication, imatinib mesylate, has been considered the most successful and selective treatment approach for many years. It inhibits the particular tyrosine kinase, BCR-ABL1. Imatinib is ineffective against leukemia stem cells (LSCs), however it usually targets the aberrant cells in leukemia (Alekseenko et al., 2022; Cavazzana-Calvo et al., 2010; Heaney and Holyoake, 2007; Holyoake, 2001; Hsieh et al., 2021; Huntly and Gilliland, 2005; Mackall et al., 2014; O'Hare et al., 2012; Patterson and Copland, 2023; Sinclair et al., 2013). Consequently, these cells are subjected to standard cancer therapies such as chemotherapy and radiation, which causes tumors to grow and recur. Therefore, by focusing on cancer stem cells, we can learn more about how normal/malignant stem cells proliferate, self-renew, and survive, which will help us better understand cancer and develop new treatments.

**TABLE 1:** TKI sensitivity of lung cancer cell lines (As shown in previous work (Phan et al., 2016)).

Cell lines	Gefitinib IC50 (μm)	Erlotinib IC50 (μm)
TKI-sensitive		
HCC827	0.1	0.1
H3255	0.1	0.1
TKI-intermediate		
H1666	1.6	1.1
HCC827C1	2.5	4.6
TKI-resistant		
H1650	12.4	16.5
H358	15.4	5.4
H1975	21.2	27
HCC827C2	27	29.7

LSCs can self-renew because the oncogene BCR-ABL1 has been shown to activate all of the key signaling pathways involved in LSC survival. However, the ability of committed progenitors to self-renew is dependent on the self-renewal characteristics of cells like HSCs and cannot be explained by the BCR-ABL1 oncogene alone (Huntly and Gilliland, 2005; Boelens, 2006; Dunn et al., 2006; Joenje and Patel, 2001; Mempel et al., 2024; Reya et al., 2001; Vera et al., 2011). Foxo, Ras, Wnt/β-catenin, Notch, Alox5, and Hedgehog (Hh) are some of the signaling pathways that may affect LSC differentiation and survival (Ahmed and Etten, 2013; Clarkson et al., 2003; Abdulmawjood et al., 2021; Deininger et al., 2000; Goldman and Melo, 2003; Huuhtanen et al., 2023).

The Musashi2 (Msi2)-Numb signaling axis is a molecular mechanism that regulates the self-renewal characteristics of LSCs and is connected to other signaling pathways such as Hh and Notch. Increased levels of numb expression could be the outcome of Msi2 deletion. This increase in Numb may reduce the number of LSCs through the important genes of the Hh and Notch signaling pathways. In order to eliminate LSCs, a detailed explanation of the significance of focusing on the Msi2-Numb signaling axis will be provided (Sinclair et al., 2013). These signalling pathways are disrupted by cancer cells, hence study in this area may offer some leukaemia therapy options (Moradi et al., 2019). Even if stronger tyrosine kinase inhibitors have been developed, certain mechanisms, specifically related to CML leukemic stem cells (CML LSC), result in recurrence, intrinsic or acquired therapeutic resistance, and the advancement of the disease. Indeed, maintenance CML LSCs in TKI-resistant patients suggest that CML LSCs contribute to drug resistance through survival mechanisms that are not entirely reliant on BCR-ABL activation. Through study and targeting of genetic alterations and molecular pathways involved in CML LSC survival in a favorable leukemic milieu and resistance to apoptosis, targeted therapy techniques seek to destroy CML LSCs in the hopes of offering a functional cure (Sinclair et al., 2013; Crews and Jamieson, 2012; Gullaksen et al., 2025; Houshmand et al., 2019; Mojtahedi et al., 2021; Soverini et al., 2021).

Thus, we aimed to present the review on CML and its progression, the recent developments on possible signalling pathways, biomarkers, natural medications, and therapeutic approaches for CML have been discussed. The detailed of the review includes CML progression, potential signaling pathways associated with CML progression and therapeutics, prognostic factors, biomarkers, and therapeutics. Therapeutics mainly covered immunotherapeutics and natural drug targets in case of CML.

## 2 CML and its progression

From their original location, cancer cells can move to different areas of the body. Anywhere blood flows, leukemia, a cancer of the bone marrow’s blood-forming tissue, can spread. Your healthcare team may be able to better plan your treatment and future care if they are aware of the typical course of CML. Often, CML advances slowly. Leukemia cells, also known as CML cells, are granulocytes that have the BCR-ABL gene and progressively begin to accumulate in the blood and bone marrow. More genetic alterations take place throughout time. Additional chromosomal abnormalities (CAs) are the term used to describe these alterations. They speed up the growth of blast cells. Consequently, the bone marrow produces additional blast cells, which then begin to proliferate in the blood. Red blood cells, white blood cells, and healthy platelets are all replaced by these cells (Albiges et al., 2022; Albiges et al., 2020; Alexander and Friedl, 2012; Ali and Sjöblom, 2009; Aranda-Anz and aldo, 2001; Ascierto et al., 2023; Bajrai et al., 2021). As the illness worsens, a person with CML may have escalating fatigue, pain on the left side around the ribcage due to a swollen spleen, and early satiety, which occurs when the spleen develops and prevents the stomach from growing when you eat. Bone discomfort, weight loss, and the recurrent infections (Clarke et al., 2016; Rinaldi and Winston, 2023).

**FIGURE 1:**
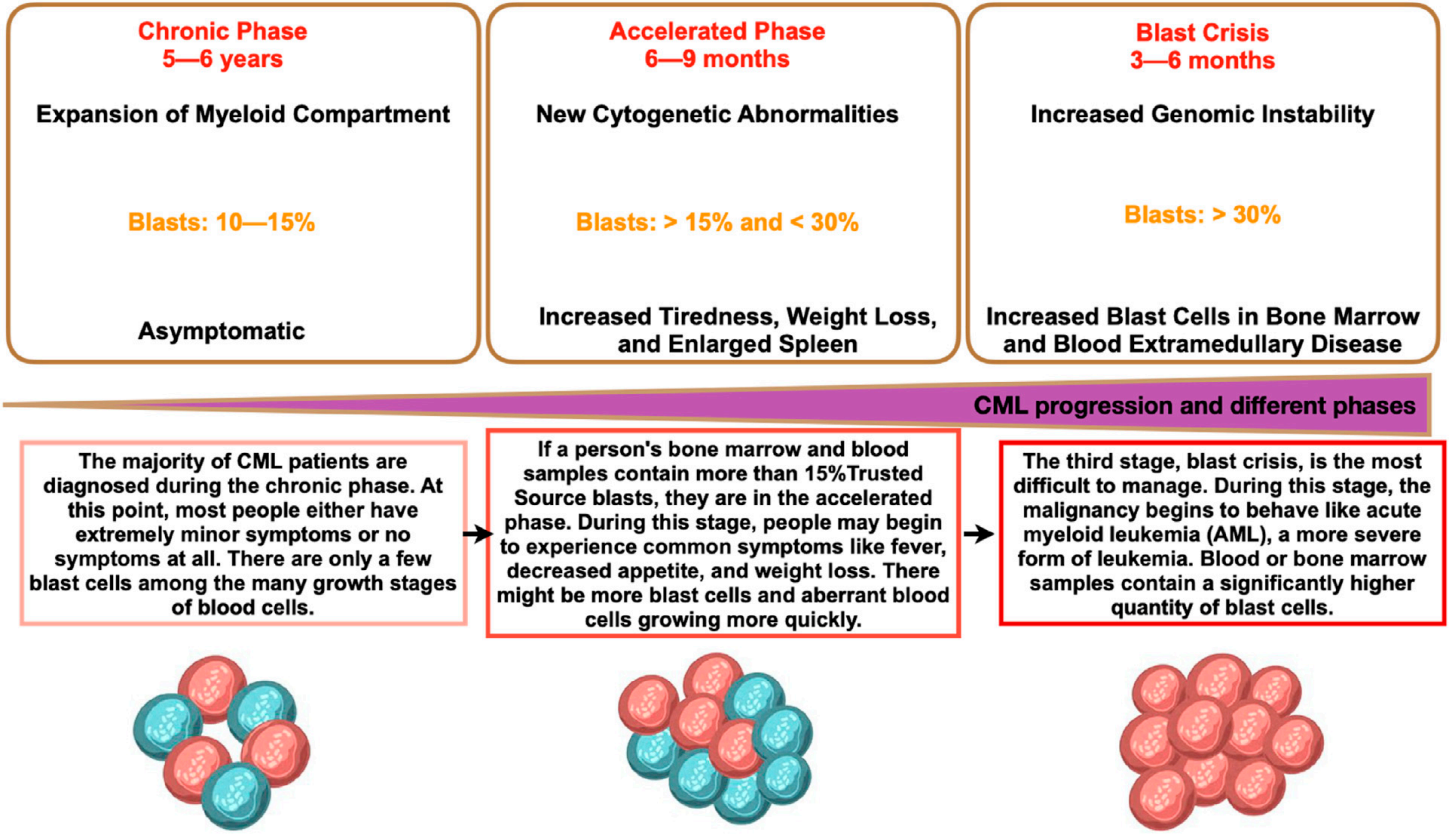
Different phases of CML and its progression. CML phases shown represent the details in each phase and the details are the time period, percentage of blast, and phase characteristics.

The two main types of myeloid leukemia are acute and chronic. Patients frequently have an underlying haematological issue, and AML advances quickly. With more than 80% of all cases, AML is the most prevalent type of leukemia. CML instances, however, are less common and account for 15% of all adult leukemia occurrences. About one instance of chronic myeloid leukemia (CML) occurs for every 100,000 people worldwide. This indicates that approximately 34,000 new cases are diagnosed annually throughout the world. About 15% of adult leukemia cases are CML. Nonetheless, CML is identified sooner in many countries with younger populations. By comparison, in the United States, there are few new cases of CML for annually (GBD, 2015 Risk Factors Collaborators, 2016; Hu et al., 2021; Lin et al., 2020). Additionally, the progression of CML is slower and takes longer before the patient reaches the rapid and blast phases than AML (Figure 1). Before discussing how it develops into myeloid sarcoma, it is imperative to examine important aspects of the pathophysiology, presentation, diagnosis, and treatment.

It is believed that a spontaneous chromosomal mutation is the source of CML, a slow-growing malignancy that starts in the bone marrow’s blood-forming cells (Horn et al., 2013; Anderson et al., 2011; Landau et al., 2013; Li et al., 2016). Myeloid sarcoma (MS), a rare tumor made of immature myeloid cells, can arise from CML and grow into a tumor mass in any part of the body other than the bone marrow. MS may arise on its own or in conjunction with another type of myeloid neoplasm (Linde et al., 2023). Within two to 3 years of the first diagnosis of chronic phase (CP), during the age of pre-tyrosine kinase inhibitor (TKI) treatment, the majority of CML cases grow before blast phase (BP). According to the research, MS in CML patients receiving TKI treatment is rare and usually only happens in isolated cases. As a result, no information is available regarding how MS affects the prognosis of people with CML. It is unclear if multiple sclerosis and medullary blood pressure have similar clinical and prognostic significance in the era of TKI therapy. Effective treatment for multiple sclerosis requires a proper diagnosis, however this is usually postponed because of the significant risk of misdiagnosis (Rinaldi and Winston, 2023; Baccarani et al., 2013; Faderl et al., 1999; Konig et al., 2008; Trinchieri, 2012).

According to studies done so far, the fact that CML exists and that patients are at risk of developing multiple sclerosis (MS) underscores the many implications that this disorder has for the practice of haematology. Even though cases are usually rare, patients have a poor prognosis, which calls for research and the use of new developments in diagnosis and therapy. It needs in-depth research because nothing is known regarding its presence throughout the medullary chronic phase or its effects during the TKI period. Additionally, it could be the initial sign of an infection before a hematopoietic system is established (Almeida et al., 2022; Ascierto et al., 2020; Atkins et al., 2022; Atkins et al., 2023; García-Gutiérrez et al., 2022; Holyoake and Helgason, 2015).

### 2.1 Different phases of CML and the aberrations

The form of treatment is mostly determined by the CML stage (CP, AP, and BP). To ascertain the stage of CML, doctors do diagnostic tests. The quantity of immature white blood cells (blasts) in the patient’s blood and bone marrow is the main factor that determines the CML phase. The majority of CML patients receive their diagnosis when the illness is in its chronic phase. Patients with chronic phase CML may or may not exhibit symptoms, have a higher white blood cell count, and typically react well to standard treatment. If treatment is not obtained, chronic phase CML will eventually proceed to either blast phase or accelerated phase CML. The quantity of immature blast cells has increased during the accelerated phase, and occasionally chromosomal alterations other than those involving the Ph chromosome will take place. Additional chromosome abnormalities in CML cells, peripheral blood basophils (a type of white blood cell) of 20% or higher, bone marrow or peripheral blood blasts of 10%–19%, and the fact that the number of CML cells grows faster in the accelerated phase—which results in symptoms like fatigue, fever, weight loss, and an enlarged spleen—are additional criteria for diagnosing accelerated phase CML. Accelerated phase CML will eventually develop into blast phase CML if treatment is not received. The blast phase resembles acute myeloid leukemia in appearance and behavior. The criteria used to diagnose blast phase CML are myeloid sarcoma, a rare form of cancer composed of myeloblasts, which are immature white blood cells, lymphoblasts (>5%), and blasts in the bone marrow or peripheral blood that are larger than or similar to 20%. Some symptoms that persons with blast phase CML may experience include fever, fatigue, shortness of breath, stomach discomfort, bone pain, enlarged spleen, low appetite and weight loss, night sweats, bleeding, and/or infections. These indicators may indicate a lymphoblastic crisis.

### 2.2 Musashi2–Numb role in chronic myeloid leukemia

The Musashi2-Numb signaling axis is essential to the development of CML, especially during the change from the chronic phase to the more aggressive blast crisis phase. By inhibiting the translation of Numb, a protein that stimulates cell differentiation and functions as a tumor suppressor, Musashi2 (MSI2), an RNA-binding protein, controls stem cell maintenance and differentiation. Higher amounts of Numb are expressed throughout the chronic phase of CML, which promotes hematopoietic cell differentiation and keeps the disease state comparatively constant. However, MSI2 expression is markedly elevated as the disease advances to blast crisis. Numb is suppressed by this rise in MSI2, which prevents differentiation and promotes the growth of undifferentiated leukemia stem cells (LSCs). These LSCs add to the aggressive character of blast crisis CML and are more resistant to traditional treatments (Moradi et al., 2019; Ito et al., 2010).

In blast crisis CML, the oncogene NUP98–HOXA9 has been found to be a major activator of MSI2 expression. This oncogene strengthens the stem-like characteristics of leukemia cells and accelerates the course of the disease by indirectly suppressing Numb by raising MSI2 levels. A possible therapeutic target, high MSI2 expression is linked to a poor prognosis and a decreased responsiveness to treatment. There are encouraging treatment prospects when the Musashi2-Numb axis is targeted. Techniques that block MSI2 or restore Numb expression may encourage leukemia cell differentiation, decrease the number of leukemia stem cells, and increase the efficacy of currently used therapies such tyrosine kinase inhibitors (Moradi et al., 2019; Ito et al., 2010; Pereira et al., 2012).

## 3 Potential signaling pathways

**FIGURE 2:**
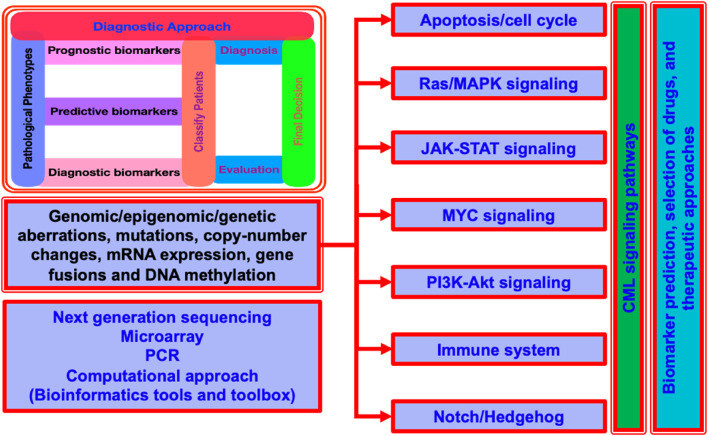
A generalized layout for the CML signaling pathways for a quick look. It display the detailed insight of aberrations at multiple levels, diagnostic approach, and the common techniques used for the understanding of CML propagation, signaling control and coordination, and the computational approaches commonly used for the understanding and exploration.

Molecular alterations in cancer genes and associated signaling pathways are used by precision medicine in cancer to direct novel cancer therapies. Small molecule inhibitors and monoclonal antibodies that target relevant cancer-related proteins have enabled successful treatments for blood cancers (e.g., imatinib for CML) and some solid tumors (e.g., trastuzumab for HER2-positive breast cancer and tamoxifen for ER-positive breast cancer). Innate limitations including drug toxicity and the emergence of acquired or *de novo* resistance mechanisms continue to be the cause of treatment failure. Understanding the benefits and drawbacks of the targeted medications now used to treat cancer is crucial. It's also critical to highlight how recent technological advancements have deepened our understanding of the molecular complexity behind resistance to cancer treatments. Furthermore, it has raised basic questions about the creation of cancer medications based on modifications to particular signaling pathways and molecular markers (Moradi et al., 2019; Maru, 2001; Melo and Deininger, 2004; Sawyers, 1999) (Figure 2). Additionally, it could be hard to understand how combination medications could prove to be a superior cancer treatment alternative than monotherapy. Furthermore, it's important to comprehend the latest therapeutic advancements that could enhance medication distribution and greatly enhance cancer patients’ clinical response and results (Abdulmawjood et al., 2021; Yip and Papa, 2021; Sanchez-Vega et al., 2018; You et al., 2023; Younes et al., 2023; Muselli et al., 2019; Ashankyty, 2025).

### 3.1 Ras and the MAP kinase pathways

There are several known relationships between Ras and Bcr-Abl. Grb-2, the adaptor molecule, has a docking site thanks to tyrosine 177 autophosphorylation. After attaching itself to the Sos protein, Grb-2 stabilizes Ras in its active GTP-bound state. Two more adaptor molecules, Shc and Crkl, can also activate Ras. Both are substrates of Bcr-Abl and bind it through their SH2 (Shc) or SH3 (Crkl) domains. However, the importance of Crkl-induced Ras activation is questioned because it appears to be restricted to fibroblasts. Moreover, direct binding of Crkl to BCR-ABL is not necessary for myeloid cell transformation.

Contrary to most other malignancies, activating mutations are uncommon, even during the disease’s blastic phase. This suggests that Ras activation is a key factor in the pathophysiology of Ph-positive leukemias. This implies that since the Ras pathway is constitutively active, no additional activating mutations are required. It is still unclear which mitogen-activated protein (MAP) kinase pathway in Ph-positive cells is downstream of Ras. The serine–threonine kinase Raf is drawn to the cell membrane when cytokine receptors, such as IL-3, are triggered when Ras is activated. Mek1/Mek2 and Erk are serine-threonine kinases that Raf uses to initiate a signaling cascade that ultimately triggers gene transcription. Although some research indicates that this route may only be activated in v-abl-transformed cells and not in BCR-ABL-transformed cells, this viewpoint has recently been challenged.

Additionally, it has been demonstrated that Bcr-Abl triggers the Jnk/Sapk pathway, which is essential for the development of cancer. Consequently, signaling from Ras may be transmitted to Gckr (germinal center kinase related) via the GTP–GDP exchange factor Rac, and subsequently down to Jnk/Sapk. There is evidence that BCR-ABL-transformed cells may also activate p38, the third pillar of the MAP kinase pathway, and that other pathways may have mitogenic potential. In either case, the signal eventually reaches the transcriptional machinery of the cell (Deininger et al., 2000; Baccarani et al., 2013; Deininger, 2013).

### 3.2 Jak-Stat pathway

The earliest evidence that the Jak-Stat pathway was involved came from earlier research on B cells that had undergone v-abl transformation. Since then, it has been demonstrated that Stat transcription factors (Stat1 and Stat5) are constitutively phosphorylated in a variety of BCR-ABL-positive cell lines and primary CML cells, and that Stat5 activation may contribute to malignant transformation. Although Stat5 has a variety of physiological functions, its effect in BCR-ABL-transformed cells appears to be primarily anti-apoptotic and involves transcriptional activation of BCL-xL. Unlike the Jak-Stat pathway, which is triggered by physiological stimuli, BCR-ABL may directly activate Stat1 and Stat5 without first phosphorylating Jak proteins. Compared to P210 BCR-ABL proteins, P190 BCR-ABL proteins seem to activate Stat6 more selectively. It would be easy to assume that this trait is related to the leukemias’ mostly lymphoblastic nature.

The role of the Ras and Jak-Stat pathways in the cellular response to growth factors may help to explain why BCR-ABL renders a number of growth factor-dependent cell lines factor independent. In some experimental settings, evidence of an autocrine loop dependent on growth factor secretion produced by BCR-ABL has been discovered. Furthermore, it was recently found that Bcr-Abl induces an autocrine loop involving G-CSF and IL-3 in early progenitor cells. Interestingly, BCR-ABL tyrosine kinase activity may also promote the production of growth factor receptors, such as the oncostatin M β receptor. Though not as much as normal progenitors, it is crucial to keep in mind that CML progenitor cells still need external growth factors for survival and proliferation throughout the chronic phase. A recent study sheds further light on this issue. FDCPmix cells transduced with a temperature-sensitive BCR-ABL mutant need few (GBD 2015 Risk Factors Collaborators, 2016)er growth inputs at the kinase permissive temperature without differentiation block. In this situation, which is comparable to chronic-phase CML, the malignant clone has a minor growth advantage while retaining nearly normal differentiation potential (Deininger et al., 2000; Baccarani et al., 2013; Deininger, 2013).

### 3.3 PI3K/AKT signaling pathway

Without PI3K activity, BCR-ABL-positive cells are unable to multiply. In order to create multimeric complexes that activate PI3K, BCR-ABL joins forces with PI3K, Cbl, and adaptor molecules Crk and Crkl. The next relevant substrate in this cascade appears to be the serine–threonine kinase Akt. This kinase was previously associated with anti-apoptotic signaling. A recent study placed Akt in the IL-3 receptor’s downstream cascade and identified the pro-apoptotic protein Bad as a critical substrate of Akt. Phosphorylated Bad is inactive because it is restricted by cytoplasmic 14-3-3 proteins and is unable to bind anti-apoptotic proteins such as BCL. This implies that the physiological IL-3 survival signal may be replicated by Bcr-Abl in a manner that is PI3K-dependent. BCR-ABL and growth factor signals activate the isothiol phosphatases Ship and Ship-2, which have somewhat different specificities. Thus, BCR-ABL appears to play a major role in phosphoinositol metabolism, which may again alter the balance in a manner similar to physiologic growth factor stimulation (Deininger et al., 2000; Baccarani et al., 2013; Deininger, 2013; Hughes et al., 2006).

### 3.4 Myc pathway

Numerous human malignancies have been shown to overexpress Myc. It is thought to work as a transcription factor, despite the fact that its target genes are mostly unknown. The SH2 domain is necessary for the Bcr-Abl-dependent activation of Myc. Overexpression of Myc partially recovers transformation-defective SH2 deletion genes, but overexpression of a dominant-negative mutant decreases transformation. As of right now, no mechanism linking Myc to the Bcr-Abl SH2 domain is understood. The signal is transduced through E2F transcription factors, cyclin-dependent kinases (CDKs), and Ras/Raf, which then activates the MYC promoter, according to data in cells that have undergone v-abl transformation. Similar results were observed with BCR-ABL-transformed murine myeloid cells. The implications of these findings for human Ph-positive cells are unknown. The effects of Myc on Ph-positive cells seem to be similar to those on other cancers. Myc could be a signal for proliferative or apoptotic processes, depending on the cellular environment. Consequently, the apoptotic arm of its dual role in CML cells is most likely being counteracted by other mechanisms, most notably the PI3 kinase pathway (O'Hare et al., 2012; Deininger et al., 2000; Baccarani et al., 2013; Deininger, 2013; Hughes et al., 2006; Eiring and Deininger, 2014; O'Hare et al., 2007).

The activity of the PI3K-AKT and Ras-ERK pathways might be adversely regulated by one another. When one pathway is chemically blocked, the cross-inhibition is released and the other pathway is effectively activated, revealing such cross-inhibition. MEK inhibitors, for instance, promote AKT activation brought on by epidermal growth factor (EGF). This procedure may entail ERK phosphorylation of GAB1 induced by EGF, which prevents PI3K from being recruited to the EGF receptor (EGFR) by GAB1. Four residues on GAB1 are phosphorylated by ERK, and substituting these locations prevents constitutively active MEK from lowering phospho-AKT levels. These ERK phospho-sites may serve to attract SHP2, which controls PI3K binding in addition to dephosphorylating the RasGAP binding sites (Mendoza et al., 2011; Adjei and Hidalgo, 2005; Aksamitiene et al., 2010; Alberghina et al., 2014).

### 3.5 Role of immune system in CML

The recent study has evaluated in detail the phenotype and function of different immune cell subsets in CML patients who wish to stop TKI therapy. The results showed that compared to patients who relapsed after 6 months of stopping imatinib, those who were in remission when TKI discontinuation occurred had more mature NK cells in their peripheral blood that displayed CD57 and CD16 antigens. Consequently, molecular relapse was associated with the proportion of NK cells that were CD56-bright (Ilander et al., 2017; Rodriguez-Mora et al., 2023). When the clinical data were examined, it was discovered that the percentage of NK cells had nothing to do with the duration of TKI treatment, previous IFN-α treatment, or the CML risk score (also known as the Sokal Score). Similar findings have also been observed by other investigations. Interestingly, NK cells, Th1 type of response, and memory T cells (both CD4^+^ and CD8^+^) were all higher in CML patients who were able to discontinue IFN-alpha monotherapy and stay in remission. Furthermore, a larger Th1 response of CD4^+^ T cells (production of TNF-α/IFN-γ) was associated with a greater number of NK cells in patients receiving TKI (Rea et al., 2017; Imagawa et al., 2015; Ilander et al., 2014). The cytokines released by antigen-presenting cells (APCs) in combination with leukemia cell-driven activation may enhance the proliferation of NK cells. Repeated stimulation may cause NK cells to develop a more mature, flexible phenotype. NK cells have the capacity to either directly destroy leukemic cells or stimulate T lymphocytes to more efficiently target leukemia (Ilander et al., 2017; Ilander et al., 2014; Ilander and Mustjoki, 2017).

The overall updates in this field indicate that the immune system is complicated and that the function of various immune cell subsets is still unclear. But when we looked at the dendritic cells (DC) from CML patients who stopped taking TKIs, we couldn't clearly see a link between the other cells and successfully stopping TKIs. It has been demonstrated that plasmocytoid DCs (pDCs) that express CD86 are associated with the tired CD8^+^ T-cell phenotype and molecular relapse. This could be one of the immunological escape mechanisms used by CML. Given these numerous discoveries, more research may be done on the connection between NK cells and pDCs. Higher NK cell counts could be a sign of potential anti-leukemia immunity, which could be mediated by both adaptive and innate immunity (Hsieh et al., 2021; Babamo et al., 2024; Qin et al., 2019; Schutz et al., 2017; Sobhani et al., 2021). The extra immune escape mechanisms that leukemic cells employ could be the subject of future research in the hunt for curative therapeutic alternatives for CML. Since NK cell modifying medications are now being tested in clinical trials for other hematological malignancies, it is imperative to assess them for CML. A higher proportion of patients may be able to cease using TKIs and move closer to a cure by employing these techniques (Huuhtanen et al., 2023; Ilander et al., 2017; Ilander et al., 2014; Ilander and Mustjoki, 2017).

## 4 Prognostic factors

In addition to the CML phase, several criteria that influence treatment choices can be used to predict a patient’s prognosis. We refer to these as prognostic factors. A less favorable prognosis is also linked to the following prognostic variables for patients with CML at the time of diagnosis (Janowski et al., 2022; Kennedy and Hobbs, 2018; Almeida et al., 2022; Ascierto et al., 2020; Atkins et al., 2022; Atkins et al., 2023; Bruck et al., 2018; Levitzki, 2013; Perez et al., 2020; Rabian et al., 2019; Zhou and Xu, 2015; Chuang et al., 2012; Sweet et al., 2013).

• CML phase: Compared to individuals with chronic phase CML, those with rapid or blast phase CML
• Age: Patients must be 60 years of age or older.
• Spleen size: Patients with an enlarged spleen in terms of size
• Platelet count: Patients with extremely high or extremely low platelet counts at diagnosis
• Blood blasts: individuals with a significant blood blast count
• Basophil counts: Patients with elevated basophil counts

Prognostic scoring methods employ several of these indicators to predict the course of treatment for patients with CML. When a patient is diagnosed with chronic phase CML, their risk profile is assessed using one of three prognostic scoring methods. These include the Hasford scoring system, the Sokal scoring system, and the European Treatment and Outcome Study for CML (EUTOS) Long-Term Survival scoring system (ELTS).

## 5 Biomarkers

Our knowledge of drug interactions is aided by the vast majority of the well-known pharmacogenomics research utilized in the medical sciences. It significantly affects how drugs are made and administered. The advancement of therapeutics depends on the widespread application of pharmacogenomics. The primary focus is on how the body’s response to drugs is influenced by genes and a complex gene system. Recent advances in clinical therapeutics have led to the discovery of novel biomarkers that assist in identifying a patient group that is more or less likely to respond to a certain medicine. By ensuring that the right prescriptions are written and that the right medication is administered at the right time in the right dose, it seeks to enhance personalized therapy. Interindividual variation in medication response is caused by a confluence of patient, environmental, and genetic factors that affect a drug’s pharmacokinetics and/or pharmacodynamics. Pharmacogenomics influences therapy efficacy, illness susceptibility, and drug development (Khan et al., 2024; Qahwaji et al., 2024; Cohen, 2008; Apellaniz-Ruiz et al., 2016; Arbitrio et al., 2018; Brown et al., 2015; Daly, 2010; Eichelbaum et al., 2006).

The molecular hallmark of CML, a rare haematopoietic malignant proliferative disease, is the Philadelphia chromosome (Ph) (Druker et al., 2001a; Druker et al., 2001b; Holyoake, 2001; Abdulmawjood et al., 2021; Faderl et al., 1999; Almeida et al., 2022; Maru, 2001; Helgason et al., 2010). Reactive oxygen species buildup and genetic instability are linked to the development of disease and are caused by an aberrant fusion gene with inappropriate kinase activity that originates on the Ph chromosome. Chromosome abnormalities and frequently changed genes are among the genetic abnormalities linked to CML in the blast phase (Abdulmawjood et al., 2021). Others, like catenin beta 1 (CTNNB1), are involved in cell adhesion; still others, like SKI like proto-oncogene (SKIL), transforming growth factor beta 1 (TGFB1), or transforming growth factor beta 2 (TGFB2), are linked to TGF-β or TNF-α pathways, like TNF-α. Some of these genes, like epidermal growth factor receptor (EGFR), tumor protein p53 (TP53), or Schmidt-Ruppin A-2 proto-oncogene (SRC), control cell apoptosis and proliferation. The role of miRNAs in CML is becoming increasingly apparent. Dysregulation of several important miRNAs, including as miRNA-451 and miRNA-21, has been linked to the development of disease states, the response to treatment, and the molecular modification of pathogenesis (Druker et al., 2001a; Druker et al., 2001b; Rinaldi and Winston, 2023; Faderl et al., 1999; Maru, 2001; Peterson et al., 2011; Thijsen et al., 1999; Warmuth et al., 1999; Wolf et al., 2013; Wylie et al., 2017; Yilmaz et al., 2015).

Assessing biomarkers is necessary to ascertain safety, effectiveness, and the mode of action. Numerous biologics and small compounds, as well as therapeutically significant cytokines, chemokines, cell surface receptors, and intracellular signaling molecules, have been discovered by prior biomarker studies.

Studies using gene expression (GE) profiling have assessed how clinically useful GE signatures are at the time of diagnosis. RNA sequencing, microarrays, and, more recently, single-cell RNA sequencing have all been used to profile patient material from different sources. However, because to variations in the cell types examined, the technologies employed, and the intrinsic complexity of genetic data interpretation, it is difficult to distill GE datasets into clinically useful markers (Krishnan et al., 2022). The current therapeutic goals include producing molecular responses profound enough to make the risks of BC transformation almost insignificant and increasing the long-term rates of treatment-free remission. As a readout of the depth of the reaction to TKI, clinical criteria for obtaining deep molecular responses have been investigated elsewhere. In essence, these guidelines suggest measuring BCR-ABL1 transcript levels every 3 months using the International Scale (IS). The degree of TKI response, in turn, is a crucial biomarker that directs patient treatment and prognosis.

An optimal biomarker would precisely identify patients who will either need to transition to alternative therapy or obtain a deep molecular response with first-line TKI, as well as those who will be able to successfully cease TKI, given the goals of current treatment. In order to facilitate early patient stratification for therapy with a first-generation TKI as opposed to a second or third-generation TKI, allosteric BCR-ABL1 inhibitor, clinical trial, or allogeneic transplant preparation, the biomarker would be instructive from the time of diagnosis and before TKI initiation. Furthermore, the ideal biomarker would detect other treatments that would improve treatment-free remissions in patients who fulfill the criteria for discontinuing TKI therapy. Finally, both low and high Human Development Index countries may have clinically reliable GE-based biomarkers that are readily accessible across centers and regions.

There are two different categories of research objectives: prognostic and mechanistic. Finding variables that, independent of treatment, can forecast an illness’s future course or result, such as survival rates, relapse risk, or disease progression, is the main goal of prognostic research. Clinicians can use these indicators to inform monitoring practices and estimate patient prognoses. A mechanistic investigation, on the other hand, aims to comprehend the fundamental biological pathways or processes that underlie the onset or course of disease. It describes the ways in which particular chemicals, genes, or biological processes influence the course of disease. For instance, increased Musashi2 expression is thought to be prognostic in CML since it is associated with poor outcomes, but it also plays a mechanistic function in increasing leukemia stem cell survival and suppressing Numb, which shows how the disease develops at the molecular level.

## 6 CML therapeutics

The presence of multipotent and controlled self-renewing hematopoietic stem cells, which leads to balanced hematopoiesis between myeloid and lymphoid lineages, is a characteristic of normal hematopoiesis. Myeloproliferative neoplasm, a rare blood malignancy characterized by rapidly proliferating and uncontrollable myeloid cells, is thought to represent the etiology in CML patients. A genetic alteration between chromosomes nine and 22 causes hematopoietic stem cells (HSCs) to create the transformative mutation known as the breakpoint cluster region gene-Abelson proto-oncogene (BCR-ABL) (Vuelta). This mutation causes the cells to become leukemic stem cells (LSCs), which leads to CML. The development of the BCR shortens chromosome 22 and destroys its vital connection to chromosome 9, leading to a disorder of hemopoietic stem cells.

This is one of the genetic distinctions between the two chromosomes. Note that the fusion of the ABL1 gene also affects chromosome 9. Although scientists have a good understanding of molecular aetiology, they still do not know what caused the genetic alteration between the two chromosomes. Nonetheless, the pathology reported has aided in determining the course of CML in afflicted patients as well as future therapy choices. Other genetic changes may transpire after BCR-ABL is established in CML CP, and the disease ultimately progresses to the deadly BC CML blastic phase. A blast crisis is a halt in the myeloid or lymphoid lineage’s maturation. Blast cells go from the bone marrow to the peripheral circulation, and LSCs produce new genetic and epigenetic abnormalities (Druker et al., 2001a; Druker et al., 2001b; Holyoake, 2001; Rinaldi and Winston, 2023; Hughes et al., 2006; Yilmaz et al., 2015; Kantarjian et al., 2006; Kantarjian et al., 2007; O'Brien et al., 2003).

The way that CML is presented is unique and impressive. Chronic anemia usually manifests as malaise, weariness, weight loss, upper left quadrant discomfort or heaviness, and in rarer cases, bleeding problems. The disease frequently progresses from the chronic stage to the accelerated stage and the final blast stage, which results in uncontrolled white cell counts in the body and the continuance of increasing symptoms. Recording CML symptoms is a step in the diagnostic procedure, and blood count information obtained from lab draws can be used to confirm these symptoms. By detecting the altered chromosomes and the BCR-ABL1 transcripts, genetic testing frequently validates the diagnosis. These are frequently extracted from bone marrow cells or peripheral blood.

Over the past few decades, CML has been treated using a variety of methods. To monitor the patient’s symptom presentation and progression, a single therapeutic approach was employed. As an alternative, chemotherapy has been used with either hydroxyurea or busulfan. Stem cell transplants, interferon-alpha (INF-a) injections, and TKIs have all been used in different situations. Combination therapy techniques have grown in popularity in recent years. To increase the efficacy of treatment regimens, this includes TKI and interferon and TKI and chemotherapy combos. In addition to a vaccine approach, immune modulation has been considered as a treatment option to elicit the desired response to leukemia activity. Despite the usage of both, there is still no known treatment for CML.

Since, the BCR-ABL translocation is present in every case of CML and serves as a target for TKI therapy as well as a diagnostic marker for the illness, CML is a “poster child” of genetically based diagnosis and treatment. The so-called chronic phase of CML is typically indicated by an increase in the number of mature myeloid cells in circulation. All instances of CML will eventually accrue new mutational events, first entering an accelerated phase and subsequently progressing to a deadly blast phase, if treatment is not received. The natural history of chronic phase sickness has changed significantly with the introduction of TKI therapy, and few patients are currently getting well while receiving treatment. Nonetheless, advanced phase disease does exist in certain patients and persons. The available treatment choices for these people are few and largely unsuccessful.

The development of diagnostic methods to forecast the course of CML and treatment options to prevent or treat it is hampered by our incomplete knowledge of the genetic “clock” that governs its advancement. The absence of mice models of CML that closely resemble human CML is one of the primary reasons for this limitation. Unlike CML blast crisis, which is primarily myeloid, the majority of mice with CML models either remain in a chronic phase or rapidly develop an acute leukemia, frequently of the lymphoid lineage. In this way, earlier research advances the field by creating a meticulously designed mouse model that accurately mimics human CML (Giotopoulos et al., 2015).

### 6.1 Cancer immunotherapy in CML

Immunotherapy is proven to be effective in treating clonal bone marrow stem cell neoplasms like CML. It is thought that patients are not cured by the existing therapy approaches, even though TKIs that target the BCR-ABL1 oncokinase are effective. New, curative therapeutic approaches, however, might result from recent developments in our knowledge of the disease’s immunobiology, such as tumor-specific antigens and immunostimulatory drugs. A small proportion of CML patients are able to discontinue treatment, despite having very few leukemia cells left, according to encouraging research. This implies that tumor cell development can be regulated by the immune system. In this work, we want to give a brief summary of the new immune system characteristics in CML patients and the changing strategies for immunotherapy-based CML control.

One kind of drug that boosts the immune system is immunotherapy. The immune system naturally produces interferon, a form of immunotherapy, but it is also possible to synthesize it in a lab. Interferon prevents cancer cells from proliferating and dividing. Interferon was thought to be the first-line treatment for patients who were not candidates for an allogeneic stem cell transplant prior to the development of TKIs. Because TKIs are more effective and have fewer side effects than interferon, interferon medicine is rarely used to treat CML these days. However, for those who are pregnant or unable to handle the side effects of TKI therapy, interferon may be a suitable alternative. The main adverse effects of interferons include mood swings, difficulty focusing and remembering things, flu-like symptoms such headaches, chills, fever, nausea, and vomiting, low red blood cell, white blood cell, and platelet counts, and difficulty concentrating and remembering things. Although these adverse effects persist as long as the patient is taking the medication, they may gradually become less severe. However, a lot of people struggle to manage these side effects on a regular basis, therefore they should discuss stopping interferon treatment with their doctor (Hsieh et al., 2021; Ureshino et al., 2020).

The innate and adaptive immune systems, which have different roles, make up the majority of the human immune system. The innate immune system is mostly composed of natural killer (NK) cells, whereas the adaptive immune system is primarily composed of T lymphocytes (T cells). Both T cells and NK cells play a major role in CML by mediating immunological responses. Because they encourage the growth of regulatory T cells (Tregs), which in turn dampen the host immune system, myeloid-derived suppressor cells (MDSCs) are also crucial. Tumor antigens (such as Wilms tumor-1) and surface receptors (such as natural killer group 2 and killer immunoglobulin-like receptor) on NK cells are significant participants in these immune system controls, even though the exact method of regulation is still unclear. Thus, we examined the latest immunological studies, concentrating on T cell and NK cell immunity in CML (Clark, 2007; Ilander et al., 2014; Ureshino et al., 2020; Owonikoko et al., 2021; Bertazzoli et al., 2000; Holmstrom and Hasselbalch, 2019).

All of these alterations returned to normal throughout imatinib therapy, and the immunoprofile began to match that of healthy controls. One set of dasatinib patients, however, appeared to be immunoactivated, as evidenced by large increases in PB’s CD8^+^, NK-, and NKT-like cells, whereas the other group appeared to be healthy controls. The latter group’s T cells contained low levels of the CD62L antigen, a sign of late memory cytotoxic lymphocytes, and significant expressions of CD57^+^, HLA-DR, and CD45RO. Their results indicate that while both TKIs have immunosuppressive properties *in vitro*, their effects on the quantity and location of immune effector cells *in vivo* are distinct and evident. In particular, a subpopulation of individuals treated with dasatinib exhibits markedly elevated immunological reactivity, necessitating careful observation (Clark, 2007; Ilander et al., 2014; Ureshino et al., 2020; Owonikoko et al., 2021; Bertazzoli et al., 2000; Holmstrom and Hasselbalch, 2019).

The interaction between natural killer (NK) cell numbers, DADI (a possible biomarker), and checkpoint-related signals like CTLA-4 and CD86 on pDC (plasmacytoid dendritic cells) is critical in the context of treatment-free remission (TFR) in CML and other malignancies. In particular, low levels of CD86+pDC may indicate effective TFR, but large levels are linked to an increased risk of relapse following TKI (tyrosine kinase inhibitor) withdrawal. NK cells have a checkpoint protein called CTLA-4, which regulates their activity. Blocking this protein can increase the cytotoxicity of NK cells (Albiges et al., 2020; Schutz et al., 2017).

### 6.2 Natural drugs/products and CML therapeutics

Chemical compounds or molecules created by living things are known as natural products. Because of their low toxicity and affordability, they are increasingly being studied in order to develop cancer medicines. Numerous lines of evidence demonstrate that many NPs, such as lignans, alkaloids, flavonoids, terpenoids, polyketides, and saponins, impede the growth of CML cells and cause apoptosis. NPs can reverse multi-drug resistance (MDR) and shift CML cells into monocyte/erythroid lineage. Here, we also present the anti-CML properties of a number of NPs. NPs are a large class of varied secondary metabolites that play a significant role in biology. Numerous organisms, including bacteria, fungi, plants, and marine life, create NPs. Because NPs are cheap and have few to no adverse effects, they are being studied as a potential treatment for infectious and cancerous disorders. Numerous NPs or NP-inspired compounds (semi-synthetic NP derivatives, synthetic compounds based on NP pharmacophores, or NP mimics) were approved, according to the records of new chemical entities (NCEs). Plant extracts, polyketides, lignans, alkaloids, flavonoids, terpenoids, and peptides were among the NPs with strong anti-CML efficacy (Faderl et al., 1999; Brown et al., 2015; Ali Abdalla et al., 2022; Childs and Carlsten, 2015; Tascilar et al., 2006; Xu et al., 2018; Wang et al., 2024).

Uncontrolled myeloid cell divisions in the bone marrow are a hallmark of chronic myeloid leukemia (CML), a hematopoietic malignancy. Chromosomes 9 and 22 reciprocally translocate to form CML [(9; 22) (q34; q11)], which ultimately leads to the formation of the bcr-abl oncogene. The “Philadelphia chromosome,” a truncated chromosome, is seen in the majority of CML patients (Ph). The constitutively active tyrosine kinase BCR-ABL is encoded by the BCR-ABL oncogene. Multiple cell proliferatory signaling pathways, including RAS, a small GTPase, mitogen activated protein kinase (MAPK), signal transducers and activator of transcription (STAT), and phosphoinositide-3-kinase (PI3K) pathways, are subsequently activated by the catalytically active kinase (Goel et al., 2023; Fleischer et al., 2016; Chuang et al., 2015).

**FIGURE 3:**
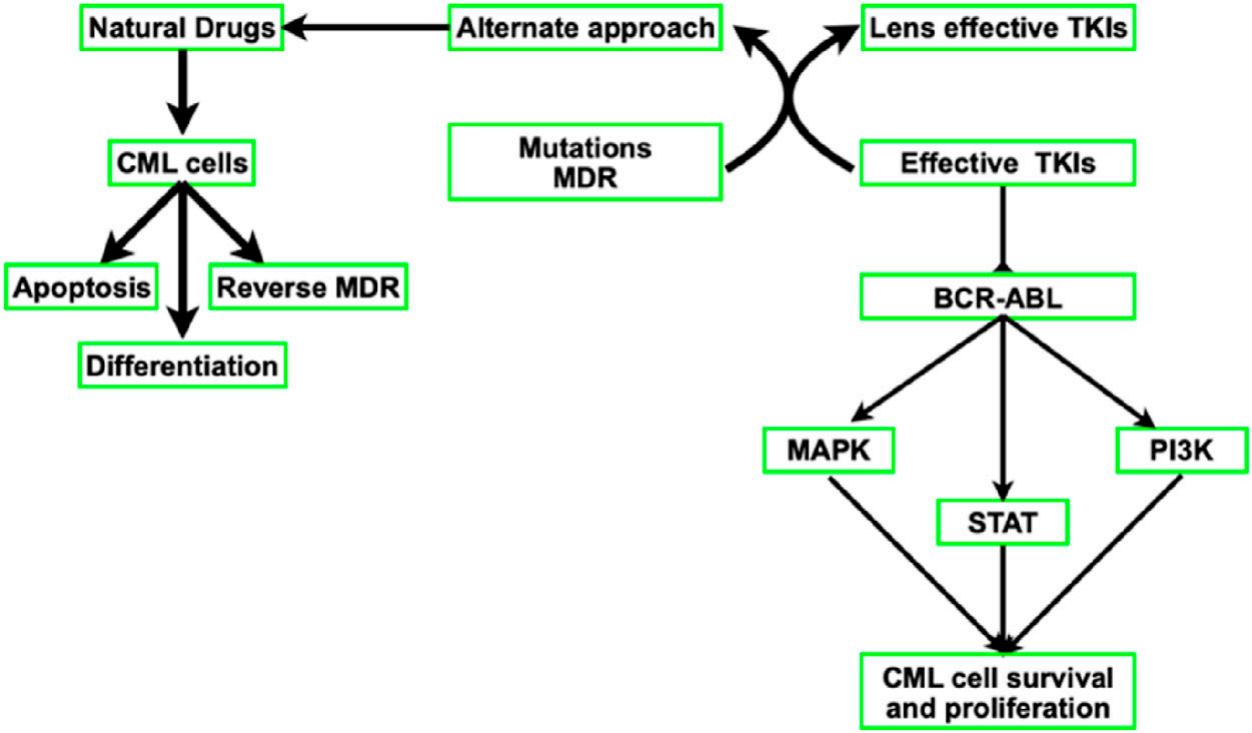
Diagram showing how natural drugs and TKIs block BCR-ABL and downregulate downstream signaling pathways.

Abl kinase targeting is unquestionably a tried-and-true method of treating CML. Imatinib, a first-generation tyrosine kinase inhibitor (TKI) that is also marketed under the names Gleevac and STI571, reduced BCR-ABL and slowed the course of CML. CML is now treated with third-generation TKIs (ponatinib), which are more effective in inhibiting BCR-ABL kinase, and second-generation TKIs like nilotinib, dasatinib, and bosutinib. The US Food and Drug Administration (FDA) approved all of these TKIs. The clinical trajectory of CML has been altered by TKIs. TKIs are less effective, nevertheless, when BCR-ABL mutations and multi-drug resistance (MDR) emerge from drug efflux brought on by p-glycoprotein overexpression. Although there is still primary or secondary resistance to TKI therapy, alternative therapeutic approaches are constantly required (Figure 3).

## 7 Future perspectives and conclusion

Here, I’ve included the most noteworthy advancements in CML research and care. Research may focus on biomarkers, drugs, especially NPs, and signaling pathways given the state of technology and available treatments for cancer diagnosis. Haematology practice is impacted in a number of ways by the existence of CML and its possible progression in affected individuals. More study is required to better understand the onset and progression of CML and determine the best therapeutic options to lower its incidence. This is especially true in situations where they could appear in soft tissue areas that are difficult to find. Additionally, in order to lower the risk of sickness development and progression during this critical period, patients in remission require closer surveillance. The majority of publications on the illness are retroactive and only include a few characteristics, making it impossible for practitioners to depend on all studies due to its rarity. Currently, this is one of the limitations. The relationship between myeloid sarcoma and chronic myeloid leukemia may become clearer with more prospective research and randomized controlled trials involving a broader patient population. Furthermore, research into biomarkers, natural medicines, and precision therapy might be necessary.

The degree, mechanisms, and co-occurrence of genetic alterations in signaling pathways that control cell proliferation, apoptosis, and cell-cycle progression differ from tumor to tumor and from tumor type to tumor, despite the fact that these alterations are commonly observed in cancer. Previous studies have used mutations, copy-number variations, mRNA expression, gene fusions, and DNA methylation to examine the processes and patterns of somatic modifications in important pathways, such as the cell cycle, Hippo, Myc, Notch, Nrf2, PI3K/AKT, RTK-RAS, TGFB signaling, p53, and B-catenin/Wnt.

## References

[B1] Abdulmawjood B. Costa B. Roma-Rodrigues C. Baptista P. V. Fernandes A. R. (2021). Genetic biomarkers in chronic myeloid leukemia: what have we learned so far? Int. J. Mol. Sci. 22 (22), 12516. 10.3390/ijms222212516 34830398 PMC8626020

[B2] Adjei A. A. Hidalgo M. (2005). Intracellular signal transduction pathway proteins as targets for cancer therapy. J. Clin. Oncol. 23 (23), 5386–5403. 10.1200/JCO.2005.23.648 15983388

[B3] Ahmed W. Etten R. A. V. (2013). Signal transduction in the chronic leukemias: implications for targeted therapies. Curr. Hematol. Malignancy Rep. 8 (1), 71–80. 10.1007/s11899-012-0150-1 PMC3886724 23307472

[B4] Aksamitiene E. Kholodenko B. N. Kolch W. Hoek J. B. Kiyatkin A. (2010). PI3K/Akt-sensitive MEK-independent compensatory circuit of ERK activation in ER-positive PI3K-mutant T47D breast cancer cells. Cell. Signal. 22 (9), 1369–1378. 10.1016/j.cellsig.2010.05.006 20471474 PMC2893265

[B5] Alberghina L. Gaglio D. Moresco R. M. Gilardi M. C. Messa C. Vanoni M. (2014). A systems biology road map for the discovery of drugs targeting cancer cell metabolism. Curr. Pharm. Des. 20 (15), 2648–2666. 10.2174/13816128113199990490 23859611

[B6] Albiges L. Tannir N. M. Burotto M. McDermott D. Plimack E. R. Barthélémy P. (2020). Nivolumab plus ipilimumab versus sunitinib for first-line treatment of advanced renal cell carcinoma: extended 4-year follow-up of the phase III CheckMate 214 trial. ESMO Open 5 (6), e001079. 10.1136/esmoopen-2020-001079 33246931 PMC7703447

[B7] Albiges L. Tannir N. M. Burotto M. McDermott D. Plimack E. R. Barthélémy P. (2022). First-line nivolumab plus ipilimumab versus sunitinib in patients without nephrectomy and with an evaluable primary renal tumor in the CheckMate 214 trial. Eur. Urol. 81 (3), 266–271. 10.1016/j.eururo.2021.10.001 34750035 PMC10202028

[B8] Alekseenko Z. Dias J. M. Adler A. F. Kozhevnikova M. van Lunteren J. A. Nolbrant S. (2022). Robust derivation of transplantable dopamine neurons from human pluripotent stem cells by timed retinoic acid delivery. Nat. Commun. 13 (1), 3046. 10.1038/s41467-022-30777-8 35650213 PMC9160024

[B9] Alexander S. Friedl P. (2012). Cancer invasion and resistance: interconnected processes of disease progression and therapy failure. Trends Mol. Med. 18 (1), 13–26. 10.1016/j.molmed.2011.11.003 22177734

[B10] Ali Abdalla Y. O. Subramaniam B. Nyamathulla S. Shamsuddin N. Arshad N. M. Mun K. S. (2022). Natural products for cancer therapy: a review of their mechanism of actions and toxicity in the past decade. J. Trop. Med. 2022, 5794350. 10.1155/2022/5794350 35309872 PMC8933079

[B11] Ali M. A. Sjöblom T. (2009). Molecular pathways in tumor progression: from discovery to functional understanding. Mol. Biosyst. 5 (9), 902–908. 10.1039/b903502h 19668850

[B12] Allavena P. Garlanda C. Borrello M. G. Sica A. Mantovani A. (2008). Pathways connecting inflammation and cancer. Curr. Opin. Genet. and Dev. 18 (1), 3–10. 10.1016/j.gde.2008.01.003 18325755

[B13] Almeida F. C. d. Berzoti-Coelho M. G. Toro D. M. Cacemiro M. d. C. Bassan V. L. Barretto G. D. (2022). Bioactive lipids as chronic myeloid leukemia’s potential biomarkers for disease progression and response to tyrosine kinase inhibitors. Front. Immunol. 13, 840173. 10.3389/fimmu.2022.840173 35493444 PMC9043757

[B14] Anderson K. Lutz C. van Delft F. W. Bateman C. M. Guo Y. Colman S. M. (2011). Genetic variegation of clonal architecture and propagating cells in leukaemia. Nature 469 (7330), 356–361. 10.1038/nature09650 21160474

[B15] Andersson A. K. Ma J. Wang J. Chen X. Gedman A. L. Dang J. (2015). The landscape of somatic mutations in infant MLL-rearranged acute lymphoblastic leukemias. Nat. Genet. 47 (4), 330–337. 10.1038/ng.3230 25730765 PMC4553269

[B16] Apellaniz-Ruiz M. Gallego C. Ruiz-Pinto S. Carracedo A. Rodríguez-Antona C. (2016). Human genetics: international projects and personalized medicine. Drug Metabolism Personalized Ther. 31 (1), 3–8. 10.1515/dmpt-2015-0032 26581075

[B17] Aranda-Anzaldo A. (2001). Cancer development and progression: a non-adaptive process driven by genetic drift. Acta Biotheor. 49 (2), 89–108. 10.1023/a:1010215424196 11450810

[B18] Arbitrio M. Di Martino M. T. Scionti F. Barbieri V. Pensabene L. Tagliaferri P. (2018). Pharmacogenomic profiling of ADME gene variants: current challenges and validation perspectives. High. Throughput 7 (4), 40. 10.3390/ht7040040 30567415 PMC6306724

[B19] Ascierto P. A. Del Vecchio M. Mandalá M. Gogas H. Arance A. M. Dalle S. (2020). Adjuvant nivolumab versus ipilimumab in resected stage IIIB-C and stage IV melanoma (CheckMate 238): 4-year results from a multicentre, double-blind, randomised, controlled, phase 3 trial. Lancet Oncol. 21 (11), 1465–1477. 10.1016/S1470-2045(20)30494-0 32961119

[B20] Ascierto P. A. Mandalà M. Ferrucci P. F. Guidoboni M. Rutkowski P. Ferraresi V. (2023). Sequencing of ipilimumab plus nivolumab and encorafenib plus binimetinib for untreated BRAF-mutated metastatic melanoma (secombit): a randomized, three-arm, open-label phase II trial. J. Clin. Oncol. 41 (2), 212–221. 10.1200/JCO.21.02961 36049147

[B21] Ashankyty I. (2025). Multifacet role of JAKs in chronic myeloid leukemia. Glob. J. Basic Sci. 1 (4), 1–5. 10.63454/jbs20000017

[B22] Atkins M. B. Jegede O. A. Haas N. B. McDermott D. F. Bilen M. A. Stein M. (2022). Phase II study of nivolumab and salvage nivolumab/ipilimumab in treatment-naive patients with advanced clear cell renal cell carcinoma (HCRN GU16-260-cohort A). J. Clin. Oncol. 40 (25), 2913–2923. 10.1200/JCO.21.02938 35442713 PMC9426835

[B23] Atkins M. B. Lee S. J. Chmielowski B. Tarhini A. A. Cohen G. I. Truong T. G. (2023). Combination dabrafenib and trametinib versus combination nivolumab and ipilimumab for patients with advanced BRAF-mutant melanoma: the DREAMseq trial-ECOG-ACRIN EA6134. J. Clin. Oncol. 41 (2), 186–197. 10.1200/JCO.22.01763 36166727 PMC9839305

[B24] Babamohamadi M. Mohammadi N. Faryadi E. Haddadi M. Merati A. Ghobadinezhad F. (2024). Anti-CTLA-4 nanobody as a promising approach in cancer immunotherapy. Cell Death Dis. 15 (1), 17. 10.1038/s41419-023-06391-x 38191571 PMC10774412

[B25] Baccarani M. Deininger M. W. Rosti G. Hochhaus A. Soverini S. Apperley J. F. (2013). European LeukemiaNet recommendations for the management of chronic myeloid leukemia: 2013. Blood 122 (6), 872–884. 10.1182/blood-2013-05-501569 23803709 PMC4915804

[B26] Bajrai L. H. Sohrab S. S. Mobashir M. Kamal M. A. Rizvi M. A. Azhar E. I. (2021). Understanding the role of potential pathways and its components including hypoxia and immune system in case of oral cancer. Sci. Rep. 11 (1), 19576. 10.1038/s41598-021-98031-7 34599215 PMC8486818

[B27] Balandrán J. C. Lasry A. Aifantis I. (2023). The role of inflammation in the initiation and progression of myeloid neoplasms. Blood Cancer Discov. 4 (4), OF1–OF13. 10.1158/2643-3230.BCD-22-0176 37052531 PMC10320626

[B28] Banjar H. R. (2018). Personalized medicine support system for chronic myeloid leukemia patients.

[B29] Bawazir A. Al-Zamel N. Amen A. Akiel M. A. Alhawiti N. M. Alshehri A. (2019). The burden of leukemia in the Kingdom of Saudi Arabia: 15 years period (1999-2013). BMC Cancer 19 (1), 703. 10.1186/s12885-019-5897-5 31315607 PMC6637507

[B30] Bertazzoli C. Marchesi E. Passoni L. Barni R. Ravagnani F. Lombardo C. (2000). Differential recognition of a BCR/ABL peptide by lymphocytes from normal donors and chronic myeloid leukemia patients. Clin. Cancer Res. 6 (5), 1931–1935. 10815918

[B31] Boelens J. J. (2006). Trends in haematopoietic cell transplantation for inborn errors of metabolism. J. Inherit. Metabolic Dis. 29 (2-3), 413–420. 10.1007/s10545-005-0258-8 16763911

[B32] Bosch F. Dalla-Favera R. (2019). Chronic lymphocytic leukaemia: from genetics to treatment. Nat. Rev. Clin. Oncol. 16 (11), 684–701. 10.1038/s41571-019-0239-8 31278397

[B33] Branford S. Wang P. Yeung D. T. Thomson D. Purins A. Wadham C. (2018). Integrative genomic analysis reveals cancer-associated mutations at diagnosis of CML in patients with high-risk disease. Blood 132 (9), 948–961. 10.1182/blood-2018-02-832253 29967129

[B34] Brown S.-A. Sandhu N. Herrmann J. (2015). Systems biology approaches to adverse drug effects: the example of cardio-oncology. Nat. Rev. Clin. Oncol. 12 (12), 718–731. 10.1038/nrclinonc.2015.168 26462128

[B35] Bruck O. Blom S. Dufva O. Turkki R. Chheda H. Ribeiro A. (2018). Immune cell contexture in the bone marrow tumor microenvironment impacts therapy response in CML. Leukemia 32 (7), 1643–1656. 10.1038/s41375-018-0175-0 29925907

[B36] Cavazzana-Calvo M. Payen E. Negre O. Wang G. Hehir K. Fusil F. (2010). Transfusion independence and HMGA2 activation after gene therapy of human β-thalassaemia. Nature 467 (7313), 318–322. 10.1038/nature09328 20844535 PMC3355472

[B37] Chandran R. K. Geetha N. Sakthivel K. M. Aswathy C. G. Gopinath P. Raj T. V. A. (2019). Genomic amplification of BCR-ABL1 fusion gene and its impact on the disease progression mechanism in patients with chronic myelogenous leukemia. Gene 686, 85–91. 10.1016/j.gene.2018.11.005 30399426

[B38] Childs R. W. Carlsten M. (2015). Therapeutic approaches to enhance natural killer cell cytotoxicity against cancer: the force awakens. Nat. Rev. Drug Discov. 14 (7), 487–498. 10.1038/nrd4506 26000725

[B39] Chuang H.-Y. Rassenti L. Salcedo M. Licon K. Kohlmann A. Haferlach T. (2012). Subnetwork-based analysis of chronic lymphocytic leukemia identifies pathways that associate with disease progression. Blood 120 (13), 2639–2649. 10.1182/blood-2012-03-416461 22837534 PMC3460686

[B40] Chuang R. Hall B. A. Benque D. Cook B. Ishtiaq S. Piterman N. (2015). Drug target optimization in chronic myeloid leukemia using innovative computational platform. Sci. Rep. 5, 8190. 10.1038/srep08190 25644994 PMC4650822

[B41] Clark R. E. (2007). Immunotherapeutic strategies in chronic myeloid leukemia. Curr. Hematol. Malig. Rep. 2 (2), 89–94. 10.1007/s11899-007-0013-3 20425356

[B42] Clarke R. T. Van den Bruel A. Bankhead C. Mitchell C. D. Phillips B. Thompson M. J. (2016). Clinical presentation of childhood leukaemia: a systematic review and meta-analysis. Arch. Dis. Child. 101 (10), 894–901. 10.1136/archdischild-2016-311251 27647842

[B43] Clarkson B. Strife A. Wisniewski D. Lambek C. L. Liu C. (2003). Chronic myelogenous leukemia as a paradigm of early cancer and possible curative strategies. Leukemia 17 (7), 1211–1262. 10.1038/sj.leu.2402912 12835715

[B44] Cohen N. (2008). Pharmacogenomics and personalized medicine. Totowa, NJ, United States: Humana Press.

[B45] Crews L. A. Jamieson C. H. M. (2012). Chronic myeloid leukemia stem cell biology. Curr. Hematol. Malignancy Rep. 7 (2), 125–132. 10.1007/s11899-012-0121-6 22467334 PMC3342507

[B46] Daly A. K. (2010). Genome-wide association studies in pharmacogenomics. Nat. Rev. Genet. 11 (4), 241–246. 10.1038/nrg2751 20300088

[B47] Deininger M. (2013). Recent advances in understanding chronic myeloid leukemia biology. Hematol. Educ. Educ. program Annu. Congr. Eur. Hematol. Assoc. 7, 139–146.

[B48] Deininger M. W. N. Goldman J. M. Melo J. V. (2000). The molecular biology of chronic myeloid leukemia. Blood 96 (10), 3343–3356. 10.1182/blood.v96.10.3343.h8003343_3343_3356 11071626

[B49] Di Giorgio C. Bellini R. Lupia A. Massa C. Urbani G. Bordoni M. (2023). The leukemia inhibitory factor regulates fibroblast growth factor receptor 4 transcription in gastric cancer. Cell Oncol. (Dordr) 47, 695–710. 10.1007/s13402-023-00893-8 37945798 PMC11090936

[B50] Druker B. J. Sawyers C. L. Kantarjian H. Resta D. J. Reese S. F. Ford J. M. (2001a). Activity of a specific inhibitor of the BCR-ABL tyrosine kinase in the blast crisis of chronic myeloid leukemia and acute lymphoblastic leukemia with the Philadelphia chromosome. N. Engl. J. Med. 344 (14), 1038–1042. 10.1056/NEJM200104053441402 11287973

[B51] Druker B. J. Talpaz M. Resta D. J. Peng B. Buchdunger E. Ford J. M. (2001b). Efficacy and safety of a specific inhibitor of the BCR-ABL tyrosine kinase in chronic myeloid leukemia. N. Engl. J. Med. 344 (14), 1031–1037. 10.1056/NEJM200104053441401 11287972

[B52] Dunn G. P. Koebel C. M. Schreiber R. D. (2006). Interferons, immunity and cancer immunoediting. Nat. Rev. Immunol. 6 (11), 836–848. 10.1038/nri1961 17063185

[B53] Eagle K. Harada T. Kalfon J. Perez M. W. Heshmati Y. Ewers J. (2022). Transcriptional plasticity drives leukemia immune escape. Blood Cancer Discov. 3 (5), 394–409. 10.1158/2643-3230.BCD-21-0207 35709529 PMC9897290

[B54] Eichelbaum M. Ingelman-Sundberg M. Evans W. E. (2006). Pharmacogenomics and individualized drug therapy. Annu. Rev. Med. 57, 119–137. 10.1146/annurev.med.56.082103.104724 16409140

[B55] Eiring A. M. Deininger M. W. (2014). Individualizing kinase-targeted cancer therapy: the paradigm of chronic myeloid leukemia. Genome Biol. 15 (9), 461. 10.1186/s13059-014-0461-8 25316524 PMC4318205

[B56] Fabbri G. Rasi S. Rossi D. Trifonov V. Khiabanian H. Ma J. (2011). Analysis of the chronic lymphocytic leukemia coding genome: role of NOTCH1 mutational activation. J. Exp. Med. 208 (7), 1389–1401. 10.1084/jem.20110921 21670202 PMC3135373

[B57] Faderl S. Talpaz M. Estrov Z. Kantarjian H. M. (1999). Chronic myelogenous leukemia: biology and therapy. Ann. Intern Med. 131 (3), 207–219. 10.7326/0003-4819-131-3-199908030-00008 10428738

[B58] Fleischer T. Chang T. T. Chiang J. H. Chang C. M. Hsieh C. Y. Yen H. R. (2016). Adjunctive Chinese Herbal Medicine therapy improves survival of patients with chronic myeloid leukemia: a nationwide population-based cohort study. Cancer Med. 5 (4), 640–648. 10.1002/cam4.627 26773538 PMC4831282

[B59] García-Gutiérrez V. Breccia M. Jabbour E. Mauro M. Cortes J. E. (2022). A clinician perspective on the treatment of chronic myeloid leukemia in the chronic phase. J. Hematol. and Oncol. 15 (1), 90. 10.1186/s13045-022-01309-0 35818053 PMC9272596

[B60] Garcia‐Manero G. Faderl S. O'Brien S. Cortes J. Talpaz M. Kantarjian H. M. (2003). Chronic myelogenous leukemia: a review and update of therapeutic strategies. Cancer 98 (3), 437–457. 10.1002/cncr.11520 12879460

[B61] GBD 2015 Risk Factors Collaborators (2016). Global, regional, and national comparative risk assessment of 79 behavioural, environmental and occupational, and metabolic risks or clusters of risks, 1990-2015: a systematic analysis for the Global Burden of Disease Study 2015. Lancet 388 (10053), 1659–1724. 10.1016/S0140-6736(16)31679-8 27733284 PMC5388856

[B62] Giotopoulos G. van der Weyden L. Osaki H. Rust A. G. Gallipoli P. Meduri E. (2015). A novel mouse model identifies cooperating mutations and therapeutic targets critical for chronic myeloid leukemia progression. J. Exp. Med. 212 (10), 1551–1569. 10.1084/jem.20141661 26304963 PMC4577832

[B63] Goel H. Kumar R. Tanwar P. Upadhyay T. K. Khan F. Pandey P. (2023). Unraveling the therapeutic potential of natural products in the prevention and treatment of leukemia. Biomed. Pharmacother. 160, 114351. 10.1016/j.biopha.2023.114351 36736284

[B64] Goldman J. M. Melo J. V. (2003). Chronic myeloid leukemia--advances in biology and new approaches to treatment. N. Engl. J. Med. 349 (15), 1451–1464. 10.1056/NEJMra020777 14534339

[B65] Golub T. R. Slonim D. K. Tamayo P. Huard C. Gaasenbeek M. Mesirov J. P. (1999). Molecular classification of cancer: class discovery and class prediction by gene expression monitoring. Science 286 (5439), 531–537. 10.1126/science.286.5439.531 10521349

[B66] Gullaksen S. E. Omsland M. Brevik M. Letzner J. Haugstvedt S. Gjertsen B. T. (2025). CML stem cells and their interactions and adaptations to tyrosine kinase inhibitors. Leuk. Lymphoma 66 (7), 1211–1220. 10.1080/10428194.2025.2466817 40598814

[B67] Hanahan D. (2022). Hallmarks of cancer: new dimensions. Cancer Discov. 12 (1), 31–46. 10.1158/2159-8290.CD-21-1059 35022204

[B68] Hanahan D. Weinberg R. A. (2011). Hallmarks of cancer: the next generation. Cell 144 (5), 646–674. 10.1016/j.cell.2011.02.013 21376230

[B69] Heaney N. B. Holyoake T. L. (2007). Therapeutic targets in chronic myeloid leukaemia. Hematol. Oncol. 25 (2), 66–75. 10.1002/hon.813 17441215

[B70] Helgason G. V. Young G. A. R. Holyoake T. L. (2010). Targeting chronic myeloid leukemia stem cells. Curr. Hematol. Malignancy Rep. 5 (2), 81–87. 10.1007/s11899-010-0043-0 20425400

[B71] Holmstrom M. O. Hasselbalch H. C. (2019). Cancer immune therapy for myeloid malignancies: present and future. Semin. Immunopathol. 41 (1), 97–109. 10.1007/s00281-018-0693-x 29987478

[B72] Holyoake D. T. (2001). Recent advances in the molecular and cellular biology of chronic myeloid leukaemia: lessons to be learned from the laboratory. Br. J. Haematol. 113 (1), 11–23. 10.1046/j.1365-2141.2001.02558.x 11328274

[B73] Holyoake T. L. Helgason G. V. (2015). Do we need more drugs for chronic myeloid leukemia? Immunol. Rev. 263 (1), 106–123. 10.1111/imr.12234 25510274

[B74] Horn M. Glauche I. Müller M. C. Hehlmann R. Hochhaus A. Loeffler M. (2013). Model-based decision rules reduce the risk of molecular relapse after cessation of tyrosine kinase inhibitor therapy in chronic myeloid leukemia. Blood 121 (2), 378–384. 10.1182/blood-2012-07-441956 23175686

[B75] Houshmand M. Simonetti G. Circosta P. Gaidano V. Cignetti A. Martinelli G. (2019). Chronic myeloid leukemia stem cells. Leukemia 33 (7), 1543–1556. 10.1038/s41375-019-0490-0 31127148 PMC6755964

[B76] Hsieh Y. C. Kirschner K. Copland M. (2021). Improving outcomes in chronic myeloid leukemia through harnessing the immunological landscape. Leukemia 35 (5), 1229–1242. 10.1038/s41375-021-01238-w 33833387 PMC8102187

[B77] Hu Y. Li Q. Hou M. Peng J. Yang X. Xu S. (2021). Magnitude and temporal trend of the chronic myeloid leukemia: on the basis of the global burden of disease study 2019. JCO Glob. Oncol. 7, 1429–1441. 10.1200/GO.21.00194 34591599 PMC8492379

[B78] Hughes T. Deininger M. Hochhaus A. Branford S. Radich J. Kaeda J. (2006). Monitoring CML patients responding to treatment with tyrosine kinase inhibitors: review and recommendations for harmonizing current methodology for detecting BCR-ABL transcripts and kinase domain mutations and for expressing results. Blood 108 (1), 28–37. 10.1182/blood-2006-01-0092 16522812 PMC1895821

[B79] Huntly B. J. Gilliland D. G. (2005). Leukaemia stem cells and the evolution of cancer-stem-cell research. Nat. Rev. Cancer 5 (4), 311–321. 10.1038/nrc1592 15803157

[B80] Huuhtanen J. Adnan-Awad S. Theodoropoulos J. Forstén S. Warfvinge R. Dufva O. (2023). Single-cell analysis of immune recognition in chronic myeloid leukemia patients following tyrosine kinase inhibitor discontinuation. Leukemia 142, 3146–17. 10.1182/blood-2023-185630 PMC10776410 37919606

[B81] Ilander M. Mustjoki S. (2017). Immune control in chronic myeloid leukemia. Oncotarget 11, 102763–102764. 10.18632/oncotarget.22279 29262520 PMC5732686

[B82] Ilander M. Kreutzman A. Rohon P. Melo T. Faber E. Porkka K. (2014). Enlarged memory T-cell pool and enhanced Th1-type responses in chronic myeloid leukemia patients who have successfully discontinued IFN-alpha monotherapy. PLoS One 9 (1), e87794. 10.1371/journal.pone.0087794 24498197 PMC3909235

[B83] Ilander M. Olsson-Strömberg U. Schlums H. Guilhot J. Brück O. Lähteenmäki H. (2017). Increased proportion of mature NK cells is associated with successful imatinib discontinuation in chronic myeloid leukemia. Leukemia 31 (5), 1108–1116. 10.1038/leu.2016.360 27890936 PMC5420794

[B84] Imagawa J. Tanaka H. Okada M. Nakamae H. Hino M. Murai K. (2015). Discontinuation of dasatinib in patients with chronic myeloid leukaemia who have maintained deep molecular response for longer than 1 year (DADI trial): a multicentre phase 2 trial. Lancet Haematol. 2 (12), e528–e535. 10.1016/S2352-3026(15)00196-9 26686407

[B85] Ito T. Kwon H. Y. Zimdahl B. Congdon K. L. Blum J. Lento W. E. (2010). Regulation of myeloid leukaemia by the cell-fate determinant Musashi. Nature 466 (7307), 765–768. 10.1038/nature09171 20639863 PMC2918284

[B86] Janowski M. Ulańczyk Z. Łuczkowska K. Sobuś A. Rogińska D. Pius-Sadowska E. (2022). Molecular changes in chronic myeloid leukemia during tyrosine kinase inhibitors treatment. Focus on immunological pathways. Onco Targets Ther. 15, 1123–1141. 10.2147/OTT.S371847 36238136 PMC9553433

[B87] Jensen H. A. Styskal L. E. Tasseff R. Bunaciu R. P. Congleton J. Varner J. D. (2013). The src-family kinase inhibitor PP2 rescues inducible differentiation events in emergent retinoic acid-resistant myeloblastic leukemia cells. PLoS ONE 8 (3), e58621. 10.1371/journal.pone.0058621 23554907 PMC3598855

[B88] Joenje H. Patel K. J. (2001). The emerging genetic and molecular basis of Fanconi anaemia. Nat. Rev. Genet. 2 (6), 446–457. 10.1038/35076590 11389461

[B89] Kantarjian H. Giles F. Wunderle L. Bhalla K. O'Brien S. Wassmann B. (2006). Nilotinib in imatinib-resistant CML and Philadelphia chromosome-positive ALL. N. Engl. J. Med. 354 (24), 2542–2551. 10.1056/NEJMoa055104 16775235

[B90] Kantarjian H. O'Brien S. Talpaz M. Borthakur G. Ravandi F. Faderl S. (2007). Outcome of patients with Philadelphia chromosome-positive chronic myelogenous leukemia post-imatinib mesylate failure. Cancer 109 (8), 1556–1560. 10.1002/cncr.22569 17342766

[B91] Kennedy J. A. Hobbs G. (2018). Tyrosine kinase inhibitors in the treatment of chronic-phase CML: strategies for frontline decision-making. Curr. Hematol. Malignancy Rep. 13 (3), 202–211. 10.1007/s11899-018-0449-7 29687320 PMC6023770

[B92] Khan B. Qahwaji R. M. Alfaifi M. S. Mobashir M. (2024). Nivolumab and ipilimumab acting as tormentors of advanced tumors by unleashing immune cells and associated collateral damage. Pharmaceutics 16 (6), 732. 10.3390/pharmaceutics16060732 38931856 PMC11207028

[B93] Konig H. Copland M. Chu S. Jove R. Holyoake T. L. Bhatia R. (2008). Effects of dasatinib on src kinase activity and downstream intracellular signaling in primitive chronic myelogenous leukemia hematopoietic cells. Cancer Res. 68 (23), 9624–9633. 10.1158/0008-5472.CAN-08-1131 19047139 PMC2786265

[B94] Krishnan V. Kim D. D. H. Hughes T. P. Branford S. Ong S. T. (2022). Integrating genetic and epigenetic factors in chronic myeloid leukemia risk assessment: toward gene expression-based biomarkers. Haematologica 107 (2), 358–370. 10.3324/haematol.2021.279317 34615339 PMC8804571

[B95] Landau D. A. Carter S. L. Stojanov P. McKenna A. Stevenson K. Lawrence M. S. (2013). Evolution and impact of subclonal mutations in chronic lymphocytic leukemia. Cell 152 (4), 714–726. 10.1016/j.cell.2013.01.019 23415222 PMC3575604

[B96] Levitzki A. (2013). Tyrosine kinase inhibitors: views of selectivity, sensitivity, and clinical performance. Pharmacol. Toxicol. 53 (1), 161–185. 10.1146/annurev-pharmtox-011112-140341 23043437

[B97] Li S. Garrett-Bakelman F. E. Chung S. S. Sanders M. A. Hricik T. Rapaport F. (2016). Distinct evolution and dynamics of epigenetic and genetic heterogeneity in acute myeloid leukemia. Nat. Med. 22 (7), 792–799. 10.1038/nm.4125 27322744 PMC4938719

[B98] Lin Q. Mao L. Shao L. Zhu L. Han Q. Zhu H. (2020). Global, regional, and national burden of chronic myeloid leukemia, 1990-2017: a systematic analysis for the global burden of disease study 2017. Front. Oncol. 10, 580759. 10.3389/fonc.2020.580759 33384954 PMC7770240

[B99] Linde M. H. Fan A. C. Köhnke T. Trotman-Grant A. C. Gurev S. F. Phan P. (2023). Reprogramming cancer into antigen-presenting cells as a novel immunotherapy. Cancer Discov. 13 (5), 1164–1185. 10.1158/2159-8290.CD-21-0502 36856575

[B100] Mackall C. L. Merchant M. S. Fry T. J. (2014). Immune-based therapies for childhood cancer. Nat. Rev. Clin. Oncol. 11 (12), 693–703. 10.1038/nrclinonc.2014.177 25348789 PMC6953414

[B101] Maru Y. (2001). Molecular biology of chronic myeloid leukemia. Int. J. Hematol. 73 (3), 308–322. 10.1007/BF02981955 11345196

[B102] Melo J. V. Deininger M. W. (2004). Biology of chronic myelogenous leukemia--signaling pathways of initiation and transformation. Hematol. Oncol. Clin. North Am. 18 (3), 545–568. 10.1016/j.hoc.2004.03.008 15271392

[B103] Mempel T. R. Lill J. K. Altenburger L. M. (2024). How chemokines organize the tumour microenvironment. Nat. Rev. Cancer 24 (1), 28–50. 10.1038/s41568-023-00635-w 38066335 PMC11480775

[B104] Mendoza M. C. Er E. E. Blenis J. (2011). The Ras-ERK and PI3K-mTOR pathways: cross-talk and compensation. Trends Biochem. Sci. 36 (6), 320–328. 10.1016/j.tibs.2011.03.006 21531565 PMC3112285

[B105] Mojtahedi H. Yazdanpanah N. Rezaei N. (2021). Chronic myeloid leukemia stem cells: targeting therapeutic implications. Stem Cell Res. Ther. 12 (1), 603. 10.1186/s13287-021-02659-1 34922630 PMC8684082

[B106] Moradi F. Babashah S. Sadeghizadeh M. Jalili A. Hajifathali A. Roshandel H. (2019). Signaling pathways involved in chronic myeloid leukemia pathogenesis: the importance of targeting Musashi2-Numb signaling to eradicate leukemia stem cells. Iran. J. Basic Med. Sci. 22 (6), 581–589. 10.22038/ijbms.2019.31879.7666 31231484 PMC6570743

[B107] Muselli F. Peyron J. F. Mary D. (2019). Druggable biochemical pathways and potential therapeutic alternatives to target leukemic stem cells and eliminate the residual disease in chronic myeloid leukemia. Int. J. Mol. Sci. 20 (22), 5616. 10.3390/ijms20225616 31717629 PMC6888542

[B108] O'Brien S. G. Guilhot F. Larson R. A. Gathmann I. Baccarani M. Cervantes F. (2003). Imatinib compared with interferon and low-dose cytarabine for newly diagnosed chronic-phase chronic myeloid leukemia. N. Engl. J. Med. 348 (11), 994–1004. 10.1056/NEJMoa022457 12637609

[B109] O'Hare T. Eide C. A. Deininger M. W. (2007). Bcr-Abl kinase domain mutations, drug resistance, and the road to a cure for chronic myeloid leukemia. Blood 110 (7), 2242–2249. 10.1182/blood-2007-03-066936 17496200

[B110] O'Hare T. Zabriskie M. S. Eiring A. M. Deininger M. W. (2012). Pushing the limits of targeted therapy in chronic myeloid leukaemia. Nat. Rev. Cancer 12 (8), 513–526. 10.1038/nrc3317 22825216

[B111] Owonikoko T. K. Park K. Govindan R. Ready N. Reck M. Peters S. (2021). Nivolumab and ipilimumab as maintenance therapy in extensive-disease small-cell lung cancer: CheckMate 451. J. Clin. Oncol. 39 (12), 1349–1359. 10.1200/JCO.20.02212 33683919 PMC8078251

[B112] Patterson S. D. Copland M. (2023). The bone marrow immune microenvironment in CML: treatment responses, treatment-free remission, and therapeutic vulnerabilities. Curr. Hematol. Malignancy Rep. 18 (2), 19–32. 10.1007/s11899-023-00688-6 36780103 PMC9995533

[B113] Pereira J. K. Traina F. Machado-Neto J. A. Duarte A. d. S. S. Lopes M. R. Saad S. T. O. (2012). Distinct expression profiles of MSI2 and NUMB genes in myelodysplastic syndromes and acute myeloid leukemia patients. Leuk. Res. 36 (10), 1300–1303. 10.1016/j.leukres.2012.06.010 22784712

[B114] Perez A. Jester G. Galili Y. El-Far A. Baidas S. (2020). Therapeutic challenges in chronic myeloid leukemia: a case-based discussion. J. Med. Cases 11 (7), 215–220. 10.14740/jmc3510 34434398 PMC8383623

[B115] Peterson L. F. Mitrikeska E. Giannola D. Lui Y. Sun H. Bixby D. (2011). p53 stabilization induces apoptosis in chronic myeloid leukemia blast crisis cells. Leukemia 25 (5), 761–769. 10.1038/leu.2011.7 21350558

[B116] Phan A. N. Hua T. N. M. Kim M. K. Vo V. T. A. Choi J. W. Kim H. W. (2016). Gallic acid inhibition of Src-Stat3 signaling overcomes acquired resistance to EGF receptor tyrosine kinase inhibitors in advanced non-small cell lung cancer. Oncotarget 7 (34), 54702–54713. 10.18632/oncotarget.10581 27419630 PMC5342374

[B117] Qahwaji R. Ashankyty I. Sannan N. S. Hazzazi M. S. Basabrain A. A. Mobashir M. (2024). Pharmacogenomics: a genetic approach to drug development and therapy. Pharmaceuticals 17 (7), 940. 10.3390/ph17070940 39065790 PMC11279827

[B118] Qin S. Xu L. Yi M. Yu S. Wu K. Luo S. (2019). Novel immune checkpoint targets: moving beyond PD-1 and CTLA-4. Mol. Cancer 18 (1), 155. 10.1186/s12943-019-1091-2 31690319 PMC6833286

[B119] Rabian F. Lengline E. Rea D. (2019). Towards a personalized treatment of patients with chronic myeloid leukemia. Curr. Hematol. Malignancy Rep. 14 (6), 492–500. 10.1007/s11899-019-00546-4 31760572 PMC6934631

[B120] Rea D. Henry G. Khaznadar Z. Etienne G. Guilhot F. Nicolini F. (2017). Natural killer-cell counts are associated with molecular relapse-free survival after imatinib discontinuation in chronic myeloid leukemia: the IMMUNOSTIM study. Haematologica 102 (8), 1368–1377. 10.3324/haematol.2017.165001 28522576 PMC6643734

[B121] Reya T. Morrison S. J. Clarke M. F. Weissman I. L. (2001). Stem cells, cancer, and cancer stem cells. Nature 414 (6859), 105–111. 10.1038/35102167 11689955

[B122] Rinaldi I. Winston K. (2023). Chronic myeloid leukemia, from pathophysiology to treatment-free remission: a narrative literature review. J. Blood Med. 14, 261–277. 10.2147/JBM.S382090 37051025 PMC10084831

[B123] Rodriguez-Mora S. Corona M. Solera Sainero M. Mateos E. Torres M. Sánchez-Menéndez C. (2023). Regular humoral and cellular immune responses in individuals with chronic myeloid leukemia who received a full vaccination schedule against COVID-19. Cancers (Basel) 15 (20), 5066. 10.3390/cancers15205066 37894433 PMC10604981

[B124] Sanchez-Vega F. Mina M. Armenia J. Chatila W. K. Luna A. La K. C. (2018). Oncogenic signaling pathways in the cancer genome atlas. Cell 173 (2), 321–337.e10. 10.1016/j.cell.2018.03.035 29625050 PMC6070353

[B125] Sawyers C. L. (1999). Chronic myeloid leukemia. N. Engl. J. Med. 340 (17), 1330–1340. 10.1056/NEJM199904293401706 10219069

[B126] Schutz C. Inselmann S. Saussele S. Dietz C. T. Mu Ller M. C. Eigendorff E. (2017). Expression of the CTLA-4 ligand CD86 on plasmacytoid dendritic cells (pDC) predicts risk of disease recurrence after treatment discontinuation in CML. Leukemia 31 (4), 829–836. 10.1038/leu.2017.9 28074067

[B127] Sinclair A. Latif A. L. Holyoake T. L. (2013). Targeting survival pathways in chronic myeloid leukaemia stem cells. Br. J. Pharmacol. 169 (8), 1693–1707. 10.1111/bph.12183 23517124 PMC3753830

[B128] Sobhani N. Tardiel-Cyril D. R. Davtyan A. Generali D. Roudi R. Li Y. (2021). CTLA-4 in regulatory T cells for cancer immunotherapy. Cancers (Basel) 13 (6), 1440. 10.3390/cancers13061440 33809974 PMC8005092

[B129] Soverini S. De Santis S. Monaldi C. Bruno S. Mancini M. (2021). Targeting leukemic stem cells in chronic myeloid leukemia: is it worth the effort? Int. J. Mol. Sci. 22 (13), 7093. 10.3390/ijms22137093 34209376 PMC8269304

[B130] Stangis M. M. Chen Z. Min J. Glass S. E. Jackson J. O. Radyk M. D. (2024). The hallmarks of precancer. Cancer Discov. 14 (4), 683–689. 10.1158/2159-8290.CD-23-1550 38571435 PMC11170686

[B131] Sweet K. Zhang L. Pinilla-Ibarz J. (2013). Biomarkers for determining the prognosis in chronic myelogenous leukemia. J. Hematol. and Oncol. 6 (1), 54. 10.1186/1756-8722-6-54 23870290 PMC3737033

[B132] Tascilar M. de Jong F. A. Verweij J. Mathijssen R. H. J. (2006). Complementary and alternative medicine during cancer treatment: beyond innocence. Oncologist 11 (7), 732–741. 10.1634/theoncologist.11-7-732 16880232

[B133] Thijsen S. Schuurhuis G. van Oostveen J. Ossenkoppele G. (1999). Chronic myeloid leukemia from basics to bedside. Leukemia 13 (11), 1646–1674. 10.1038/sj.leu.2401565 10557038

[B134] Trinchieri G. (2012). Cancer and inflammation: an old intuition with rapidly evolving new concepts. Immunology 30 (1), 677–706. 10.1146/annurev-immunol-020711-075008 22224761

[B135] Ureshino H. Shindo T. Kimura S. (2020). Role of cancer immunology in chronic myelogenous leukemia. Leuk. Res. 88, 106273. 10.1016/j.leukres.2019.106273 31765938

[B136] Vera J. Rateitschak K. Lange F. Kossow C. Wolkenhauer O. Jaster R. (2011). Systems biology of JAK-STAT signalling in human malignancies. Prog. Biophysics Mol. Biol. 106 (2), 426–434. 10.1016/j.pbiomolbio.2011.06.013 21762720

[B137] Wang Z. Liu Z. Qu J. Sun Y. Zhou W. (2024). Role of natural products in tumor therapy from basic research and clinical perspectives. Acta Mater. Medica 3 (2). 10.15212/amm-2023-0050

[B138] Warmuth M. Danhauser-Riedl S. Hallek M. (1999). Molecular pathogenesis of chronic myeloid leukemia: implications for new therapeutic strategies. Ann. Hematol. 78 (2), 49–64. 10.1007/s002770050473 10089019

[B139] Wolf A. Eulenfeld R. Gäbler K. Rolvering C. Haan S. Behrmann I. (2013). JAK2-V617F-induced MAPK activity is regulated by PI3K and acts synergistically with PI3K on the proliferation of JAK2-V617F-positive cells. JAK-STAT 2 (3), e24574. 10.4161/jkst.24574 24069558 PMC3772110

[B140] Wylie A. A. Schoepfer J. Jahnke W. Cowan-Jacob S. W. Loo A. Furet P. (2017). The allosteric inhibitor ABL001 enables dual targeting of BCR–ABL1. Nature 543 (7647), 733–737. 10.1038/nature21702 28329763

[B141] Xu C. Chen Y. P. Du X. J. Liu J. Q. Huang C. L. Chen L. (2018). Comparative safety of immune checkpoint inhibitors in cancer: systematic review and network meta-analysis. BMJ 363, k4226. 10.1136/bmj.k4226 30409774 PMC6222274

[B142] Yilmaz M. Abaza Y. Jabbour E. (2015). Selecting the best frontline treatment in chronic myeloid leukemia. Curr. Hematol. Malignancy Rep. 10 (2), 145–157. 10.1007/s11899-015-0254-5 25921387 PMC5459321

[B143] Yip H. Y. K. Papa A. (2021). Signaling pathways in cancer: therapeutic targets, combinatorial treatments, and new developments. Cells 10 (3), 659. 10.3390/cells10030659 33809714 PMC8002322

[B144] You M. Xie Z. Zhang N. Zhang Y. Xiao D. Liu S. (2023). Signaling pathways in cancer metabolism: mechanisms and therapeutic targets. Signal Transduct. Target Ther. 8 (1), 196. 10.1038/s41392-023-01442-3 37164974 PMC10172373

[B145] Younes S. Ismail M. A. Al-Jurf R. Ziyada A. Nasrallah G. K. Abdulrouf P. V. (2023). Management of chronic myeloid leukaemia: current treatment options, challenges, and future strategies. Hematology 28 (1), 2196866. 10.1080/16078454.2023.2196866 37078896

[B146] Zhou H. Xu R. (2015). Leukemia stem cells: the root of chronic myeloid leukemia. Protein and Cell 6 (6), 403–412. 10.1007/s13238-015-0143-7 25749979 PMC4444810

